# Impulsive Personality Traits Mediate the Relationship Between Attention-Deficit/Hyperactivity Disorder Symptoms and Psychiatric Comorbidity among Patients with Severe Alcohol Use Disorder

**DOI:** 10.1080/15504263.2021.1944711

**Published:** 2021-07-27

**Authors:** Laura Brandt, Frances R. Levin, Dominik Kraigher

**Affiliations:** a Division on Substance Use Disorders, New York State Psychiatric Institute, New York, New York, USA; b Department of Psychiatry, Columbia University Irving Medical Center, New York, New York, USA; c Anton Proksch Institut, Vienna, Austria

**Keywords:** Alcohol use disorder, attention-deficit hyperactivity disorder, impulsive personality traits, mediation, psychiatric comorbidity

## Abstract

Objective

Attention-deficit/hyperactivity disorder (ADHD) is an established risk factor for developing alcohol use disorder (AUD), and AUD-ADHD comorbidity is associated with additional psychiatric diagnoses. Several lines of evidence support the role of impulsivity as a pathway of these relationships; however, impulsivity is not a unitary construct. Thus, we sought to explore whether separate aspects of impulsivity may explain the relationship between ADHD symptoms and psychiatric comorbidity among inpatients (*N* = 136) with AUD. **Methods:** We assessed ADHD symptoms (childhood ADHD [Wender Utah Rating Scale], adult ADHD [Adult ADHD self-report scale]), health-related quality of life (HRQL; EQ-5D-5L), psychiatric comorbidity (Mini International Neuropsychiatric Interview), and impulsive personality traits (Urgency, Premeditation, Perseverance, Sensation seeking [UPPS] scale). **Results:** 19% of patients screened positive in the retrospective assessment of childhood ADHD, and 17% for adult ADHD. Participants reported moderate levels of problem severity in the HRQL dimensions, and 65% had ≥1 current psychiatric disorders other than AUD and ADHD. Multiple mediation indicated that there was a significant direct effect of childhood ADHD symptoms on psychiatric comorbidity (*β* = 0.224, 95% CI [0.080, 1.114]), and indirect effects of both reacting impetuously when experiencing negative emotions (negative urgency; *β* = 0.999, 95% CI [0.043, 0.461]) and the tendency to not finish tasks (lack of perseverance; *β* = 0.075, 95% CI [0.002, 0.297]). **Conclusions:** The subcomponents of impulsivity to react rashly when experiencing negative emotions and the tendency to not persist in activities seem to contribute to the relationship between ADHD symptoms (particularly those in childhood) and psychiatric comorbidity among patients with severe AUD.

Alcohol use disorder (AUD), characterized by an impaired ability to stop or control alcohol use despite adverse social, occupational, or health consequences, is among the most common mental health problems (Forouzanfar et al., 2015) and contributes significantly to the global morbidity and mortality burden (Grant et al., 2015; Griswold et al., 2018; Hay et al., 2017). Attention-deficit hyperactivity disorder (ADHD) is a frequent comorbidity in adults seeking treatment for AUD with prevalence rates ranging from 5% to 22% (van de Glind et al., 2014). In patients with AUD, ADHD is associated with an earlier age of first substance use, increased AUD severity, more psychiatric diagnoses, a greater likelihood of attempted suicide, and more hospitalizations (Daurio et al., 2018; Ercan, 2003; Ibrahim et al., 2015; Moura et al., 2013; Young et al., 2020).

ADHD is an established risk factor for developing AUD (Charach et al., 2011; Lee et al., 2011; Squeglia et al., 2016). Several biological and cognitive differences in individuals with and without ADHD may contribute to the connection between these two disorders (Maxwell, 2013), and impulsivity seems to be a key commonality between adolescent behavior problems and adult alcohol problems. The mechanisms involved in impulsive behavior such as impaired control over alcohol use despite negative consequences in AUD and impaired social and work life due to difficulties in waiting one’s turn and failure to inhibit inappropriate responses associated with ADHD show remarkable similarities and overlap in both disorders.

Progress in understanding the relationship between impulsivity and alcohol use/AUD has likely been slowed by the imprecise use of the term “impulsivity” and inconsistencies in conceptualization (Dick et al., 2010; Whiteside & Lynam, 2001). In an attempt to identify and separate distinct personality facets that have been previously lumped together under the generic term impulsivity, Whiteside and Lynam (2001) constructed a scale based on a factor analysis of existing measures of impulsivity which assesses four distinct facets of personality associated with impulsive behavior: (1) negative urgency defined as acting rashly when experiencing extreme negative emotions; (2) (lack of) perseverance defined as the tendency to not finish tasks; (3) (lack of) planning/premeditation defined as acting without thinking; and (4) sensation seeking defined as behavior tendencies of trying new and exciting activities or sensations. These four facets are not considered to be variations of impulsivity but are conceptualized as discrete psychological processes that lead individuals to engage in behavior seemingly without a proper appreciation of the potential negative consequences. More recent research has identified a positive emotion variant of urgency (positive urgency; Cyders et al., 2007). The size of the relationship between impulsivity based on the UPPS-P model and alcohol use varies by impulsivity trait (Coskunpinar et al., 2013). For example, drinking quantity is most strongly predicted by lack of perseverance, while drinking problems are highly related to negative and positive urgency and alcohol dependence to negative urgency and lack of planning.

Separate impulsivity facets may differentially contribute to the association between ADHD and AUD. For example, findings from a non-clinical student sample suggest that lack of perseverance and sensation seeking are the facets of impulsivity that explain the relation between ADHD symptoms and alcohol use (Roberts et al., 2014). In a sample of adults who had been initially diagnosed with ADHD in childhood and were prospectively followed, urgency mediated the relationship between childhood ADHD and number of alcohol problems in adulthood (Pedersen et al., 2016). Consistent with this finding, another study (Daurio et al., 2018) found that urgency mediated the relationship of adult ADHD symptoms with AUD severity. However, these results are limited to American samples and to individuals with either a formal diagnosis of ADHD in childhood without verification of persistence into adulthood (Pedersen et al., 2016), or those with adult ADHD measured with the Conners’ Adult ADHD Rating Scales (CAARS; Daurio et al., 2018; Roberts et al., 2014). CAARS results are based on an individual’s current functioning and cannot be used to establish the childhood onset of symptoms, which is necessary for diagnosis (Kooij et al., 2010).

Moreover, it has been shown that ADHD is associated with a higher prevalence of comorbid disorders in individuals with AUD (Moura et al., 2013; Roncero et al., 2019; Young et al., 2020) and in other substance use disorder (SUD) samples (van Emmerik-van Oortmerssen et al., 2014). Several facets of impulsivity have been identified as risk factors for ADHD, suicide risk, mood disorders, anxiety disorders and SUDs among individuals with gambling disorder (Grall-Bronnec et al., 2012). However, to our knowledge, the relationships between ADHD, specific impulsivity traits and psychiatric comorbidity have not been studied in AUD patients.

Given the heightened prevalence of additional psychiatric diagnoses among individuals with AUD-ADHD comorbidity, this study sought to explore whether separate facets of impulsivity may explain the relationship between ADHD screening status and psychiatric comorbidity. We hypothesized that underlying pathways of impulsive behavior (specifically urgency and lack of perseverance; Grall-Bronnec et al., 2012) would partially account for the relationship between ADHD symptoms and psychiatric comorbidity among inpatients with AUD.

## Methods

### Recruitment

Patients were recruited at the Anton Proksch Institute (API), a large inpatient clinic for addiction treatment in Vienna, Austria. Participation in the study was voluntary and anonymous. There was a complete discussion of the study with potential participants and written informed consent was obtained after this discussion. The study was conducted in accordance with the Declaration of Helsinki (World Medical Association, 2013) and the University of Vienna’s Ethics Committee approved all study procedures.

Inpatients were eligible to participate in the study if they were at least 18 years of age, had a primary International Statistical Classification of Diseases and Related Health Problems 10th revision (ICD-10; World Health Organization, 2004) diagnosis of AUD (F10.10 or F10.20) listed in their chart, had sufficient German language skills, and signed written informed consent. Exclusion criteria included acute intoxication, acute psychotic episode, insufficient spatial or temporal orientation, and/or the inability to understand or to repeat/recall the study information.

### Participants

A total of 136 participants (27.9% female), aged 22–77 years, took part in this study. No patient who expressed interest in the study had to be excluded based on the criteria listed above. On average, participants endorsed 8.76 symptoms (*SD* = 1.81) Diagnostic and Statistical Manual of Mental Disorders 5th edition (DSM-5; American Psychiatric Association, 2013) criteria for AUD in the past 12 months. Eight participants (5.9%) endorsed four to five criteria (indicative of moderate AUD severity), but most (94.1%) had severe current AUD (i.e., they endorsed 6 or more DSM-5 AUD symptoms). Socio-demographic characteristics of the sample are displayed in Table 1.

**Table 1.** Characteristics of the total sample and by ADHD screening status.

	ADHD screening status			
	No history of ADHD^a^	Childhood ADHD^b^	Adult ADHD^c^	Total
	(*N* = 87)	(*N* = 26)	(*N* = 23)	(*N* = 136)
Sex, *N* (%)				
Male	56 (64.4)	23 (88.5)	19 (82.6)	98 (72.1)
Female	31 (35.6)	3 (11.5)	4 (17.4)	38 (27.9)
Marital status, *N* (%)				
Single/separated/divorced/widowed	62 (71.3)	21 (80.8)	20 (87.0)	103 (75.7)
Married/co-habiting	25 (28.7)	5 (19.2)	3 (13.0)	33 (24.3)
Highest educational attainment, *N* (%)				
Compulsory education	9 (10.3)	3 (11.5)	5 (21.7)	17 (12.5)
Vocational training	37 (42.5)	18 (69.2)	11 (47.8)	66 (48.5)
Secondary education	19 (21.8)	3 (11.5)	3 (13.0)	25 (18.4)
University degree	20 (23.0)	1 (3.8)	3 (13.0)	24 (17.6)
Employment status, *N* (%)				
Unemployed	54 (62.1)	16 (61.5)	17 (73.9)	87 (64.0)
Employed	32 (36.8)	10 (38.5)	6 (26.1)	48 (35.3)
Impulsivity (UPPS), *M* (SD)				
Negative urgency	29.7 (6.76)	33.7 (5.94)	36.2 (6.61)	31.6 (7.04)
Lack of perseverance	17.3 (5.25)	19.8 (2.99)	22.7 (5.36)	18.7 (5.31)
Lack of premeditation	22.3 (5.44)	23.3 (5.16)	25.1 (5.18)	23.0 (5.41)
Sensation seeking	31.4 (8.08)	35.3 (7.36)	35.7 (7.54)	32.8 (8.05)
Current psychiatric comorbidities				
Number of comorbidities, *M* (SD)	1.09 (1.25)	1.69 (1.52)	1.57 (1.31)	1.29 (1.33)
≥1 comorbidity, *N* (%)	50 (58.1)	21 (80.8)	17 (73.9)	88 (65.2)

^a^WURS-k Score < 30; ^b^WURS-k score ≥ 30 and three or less marks in the darkly shaded boxes within part A of the ASRS-v1.1; ^c^WURS-k score ≥ 30 and four or more marks in the darkly shaded boxes within part A of the ASRS-v1.1.

### Procedure and measures

A standardized and structured interview was completed in a single session (approximately 90 min without breaks to keep study conditions as equal as possible for all participants). Participants were compensated for the time spent in the study with €10. Besides socio-demographic information, the following variables were assessed.

### Alcohol use disorder severity

The DSM-5 checklist for AUD was used to determine the severity of the patients’ primary AUD diagnosis during the past 12 months. Endorsement of 2–3 criteria is indicative of mild AUD, endorsement of 4–5 symptoms is indicative of moderate AUD, and endorsement of six or more symptoms is indicative of severe AUD.

### ADHD

The Adult ADHD Self-Report Scale (ASRS-v1.1; Adler et al., 2003) is an 18-item self-report inventory based on DSM-IV-TR criteria, and was used to rate current ADHD symptoms using a 5-point Likert severity scale. If four or more marks appear in the darkly shaded boxes within Part A, the participant has symptoms highly consistent with ADHD (World Health Organization, 2012). The validity of the short version of the scale, consisting of six items (part A of the long version), is reported as acceptable among AUD patients with a sensitivity (true positive rate; i.e., the proportion of individuals who have ADHD and are correctly identified as having ADHD) of ≥ 80% (Daigre et al., 2015; Reyes et al., 2019; van de Glind et al., 2013). If used in combination with the WURS-k (see below), the psychometric properties are substantially improved (Daigre et al., 2015).

The Wender Utah Rating Scale-deutsche Kurzform (WURS-k) is a widespread standardized instrument with 25 items rated on a 5-point scale, and was used for the retrospective assessment of childhood ADHD symptoms in adults (Retz-Junginger et al., 2002). A score of ≥ 30 suggests prevalence of childhood ADHD symptoms. If the symptoms do not persist into adulthood, the disorder is considered to be in partial remission (Rösler et al., 2009).

Patients were also asked if they had ever received ADHD treatment, and if so what kind of treatment.

### Health related quality of life

Health related quality of life (HRQOL) represents the effects of an illness upon the physical, mental, and social dimensions of an individual’s well-being. The EuroQol-5 Dimension (EQ-5D) is a short questionnaire that consists of a description and a valuation of HRQOL as a summarized single index score reflecting individual preferences for different health states (The EuroQolGroup, 1999). The EQ-5D has shown to be valid in patients with AUD (Gunther et al., 2007).

For the purpose of this study, we used the EQ-5D-5L version (The EuroQolGroup, 2020), with improved sensitivity and reduced ceiling effects as compared to the previous EQ-5D-3L. The scale covers five dimensions of health: mobility, self-care, usual activities, pain/discomfort, and anxiety/depression. Each of the dimensions is divided into five levels of perceived problems (1—no problem to 5—unable to/extreme problems). The questionnaire comprises an additional visual analogue scale (VAS) on which patients rate their perceived current health from 0 (the worst imaginable health) to 100 (the best imaginable health).

### Psychiatric comorbidities

Psychiatric comorbidities were assessed using the Mini International Neuropsychiatric Interview (MINI; Sheehan et al., 2000), a brief structured interview for major Axis I psychiatric disorders in DSM-IV and ICD-10 including mood disorders, anxiety disorders, SUDs, psychotic disorders, and eating disorders, as well as antisocial personality disorder (ASPD). The validity and reliability of the MINI are similar to the Structured Clinical Interview for DSM (SCID; First & Gibbon, 2004) and the Composite International Diagnostic Interview (CIDI; Nelson, 1999).

### Impulsivity/impulsive behavior

Given that impulsivity is increasingly recognized as a multidimensional construct (Coskunpinar et al., 2013; Dick et al., 2010), we examined separate impulsivity-related constructs based on the UPPS model of impulsivity (Whiteside & Lynam, 2001). The validity of the UPPS conceptualization of impulsivity has been tested in individuals seeking help for their alcohol use, those with borderline personality disorder, and pathological gamblers (Whiteside et al., 2005). We used the validated German version of the UPPS scale (Schmidt, et al., 2008) including 45 statements rated on a 4-point scale.

### Data analysis

Between-group analyses with ADHD screening status (no history of ADHD, ADHD symptoms in childhood only, ADHD persistence in adulthood) as the independent variable were conducted using analysis of variance (ANOVA). Welch’s statistic was reported if the assumption of homogeneity of variances was not met. In the case of significant ANOVAs, Tukey pairwise comparisons identified specific group differences. Other between-group analyses were conducted using tests that were appropriate for the dependent variable (i.e., independent *t*-test for continuous outcome variables and Chi-Square test for categorical variables).

Next, we tested the hypothesis that impulsivity mediates the relationship between ADHD screening status and psychiatric comorbidity (i.e., number of current psychiatric disorders according to the MINI including mood and anxiety disorders, SUDs other than AUD, and ASPD). A multiple mediation model was built to test whether impulsivity (via UPPS subscales) mediated the relationship of ADHD screening status and number of psychiatric comorbidities. In mediation analysis, the total effect (i.e., the effect of the independent on the dependent variable) can be broken down into two parts: the direct and indirect effect. The direct effect is the effect of the independent variable (ADHD screening status) on the outcome (number of psychiatric comorbidities) absent the mediators. The indirect pathway is the effect of the independent variable on the outcome that works through the mediators (impulsivity traits).

We performed these analyses using Jamovi software, version 1.0.7.0 (Gallucci, 2019; R Core Team, 2019; Rosseel, 2012; The jamovi project, 2020). All coefficients were estimated using the maximum likelihood method, and betas were obtained as standardized parameters of the path model. Standardized beta coefficients have standard deviations as their units so that variables can be compared to each other. They compare the strength of the effect of each individual independent variable to the dependent variable. The higher the absolute value of the beta coefficient, the stronger the effect. Confidence intervals were estimated with bootstrap percentile method, as suggested for smaller samples, with a 1,000-resampling iterations process, bootstrapping the full mediation model for each level of the moderator. Mediation analysis controlled for sex and age.

## Results

### ADHD screening status

Twenty-six patients (19.1% of the sample) screened positive in the retrospective assessment of childhood ADHD (i.e., exceeded the cutoff for the WURS-k) but symptoms of ADHD were no longer present (i.e., these participants did not exceed the cutoff for the ASRS-v1.1). ADHD symptoms persisted in adulthood in 23 patients (16.9% of the sample); i.e., these patients exceeded the cutoff for childhood ADHD (WURS-k) and adult ADHD (ASRS-v1.1). Based on WURS-k and ASRS-v1.1 scores, it was possible to classify participants into three ADHD groups (see Table 1). Fourteen patients (10.3% of the sample) met criteria on the ASRS-v1.1 but not the WURS-k. However, with an AUD sample, motivational and cognitive deficits resulting from prolonged substance use can mimic adult ADHD symptoms. In addition, several symptoms must have been present prior to age 12 for tentative ADHD diagnosis. Thus, these patients were classified in the “no history of ADHD” group—along with those who met neither cutoff—for the purpose of this study.

There was a significant association between sex and ADHD group classification, *Χ*^2^(2) = 7.30, *p* = .026. Women were less frequently classified in childhood and adult ADHD groups than men. In addition, there were differences between the three ADHD groups in several aspects of impulsivity. Table 2 indicates bivariate correlations between adult ADHD symptoms, childhood ADHD symptoms, and impulsivity traits. Negative urgency differed by ADHD group, *F*(2,133) = 10.48, *p* < .001, partial *η*^2^ = .136. Compared to participants without a history of ADHD, both those with a history of childhood ADHD symptoms (mean difference = 3.97, *p* = .022) and those with adult ADHD symptoms (mean difference = 6.49, *p* < .001) had higher negative urgency scores. Similarly, there was a significant association between ADHD group classification and lack of perseverance, *F*(2,133) = 12.09, *p* < .001, partial *η*^2^ = .154. However, only the difference between participants without a history of ADHD and those with adult ADHD symptoms reached significance (mean difference = 5.46, *p* < .001). Moreover, there was an overall difference between the three ADHD groups in sensation seeking, *F*(2,133) = 4.35, *p* = .015, partial *η*^2^ = .061. However, post-hoc comparisons between the three groups did not reach significance.

**Table 2.** Bivariate correlations between adult ADHD symptoms (Part A of the ASRS-v1.1 sum score), childhood ADHD symptoms (WURS-k sum score), impulsivity traits (urgency, premeditation, perseverance, sensation seeking; UPPS subscale sum scores), and number of current psychiatric diagnoses (MINI).

	*M*	*SD*	1	2	3	4	5	6	7
1. Adult ADHD symptoms	9.63	4.37	1	.534**	.539**	.293**	.502**	.194*	.314**
2. Childhood ADHD symptoms	24.87	15.80	–	1	.507**	.290**	.396**	.300**	.462**
3. Urgency	31.58	7.04	–	–	1	.294**	.390**	.186*	.380**
4. Premeditation	22.96	5.41	–	–	–	1	.460**	.311**	.102
5. Perseverance	18.69	5.31	–	–	–	–	1	.006	.298**
6. Sensation seeking	32.85	8.05	–	–	–	–	–	1	.046
7. Number of current psychiatric comorbidities	1.29	1.33	–	–	–	–	–	–	1

*Note.* ADHD = attention-deficit/hyperactivity disorder; ASRS = Adult ADHD self-report scale; WURS-k = Wender Utah Rating Scale – deutsche Kurzform; MINI = Mini International Neuropsychiatric Interview.

*p < .05, **p < .01.

Five participants reported that they had been treated for ADHD in the past, and two of those reported that they had received ADHD medication as part of this treatment. Four of those participants screened positive for adult ADHD in the current study, and one for childhood ADHD only.

### Health-related quality of life

Table 3 depicts problems participants indicated regarding their HRQL, and the level of these problems among those who reported any. The two dimensions that affected participants most were pain/discomfort and anxiety/depression. However, most patients reported relatively low levels of problem severity in these dimensions. Overall, only one participant reported that problems doing usual activities (e.g., work, study, housework, family, or leisure activities) were so severe that it was no longer possible to carry them out.

**Table 3.** Numbers and proportions reporting problems within EQ-5D dimensions.

		Dimension				
		Mobility	Self-care	Usual activities	Pain/discomfort	Anxiety/depression
*N* (%) of sample reporting any problems		29 (21.2)	6 (4.4)	23 (16.9)	68 (49.6)	58 (42.6)
Level, *N* (%)^a^						
	Slight	16 (55.2)	2 (33.3)	16 (69.6)	39 (58.2)	35 (60.3)
	Moderate	9 (31.0)	3 (50.0)	5 (21.7)	19 (28.4)	17 (29.3)
	Severe	4 (13.8)	1 (16.7)	1 (4.3)	9 (13.4)	6 (10.3)
	Unable to/extreme	0 (0.0)	0 (0.0)	1 (4.3)	0 (0.0)	0 (0.0)

^a^*N* (%) of those who reported any problems.

The separate EQ-5D VAS rating indicated that participants perceived their current overall health as relatively good, with a mean score of 75.77 (*SD* = 14.20). Participants who reported at least one problem in the EQ-5D dimensions indicated a significantly worse current overall health (*M* = 71.39, *SD* = 14.54) than those who reported no problems (*M* = 83.63, *SD* = 9.65), *t*(133) = 5.23, *p* < .001, *d* = 0.99.

There was no significant association between ADHD screening status and overall severity of problems regarding HRQL, *F*(2,132) = 2.23, *p* = .112, or between ADHD screening status and current overall EQ-5D VAS rating, *F*(2,133) = 1.37, *p* = .258.

### Psychiatric comorbidities other than ADHD

**Figure 1.**
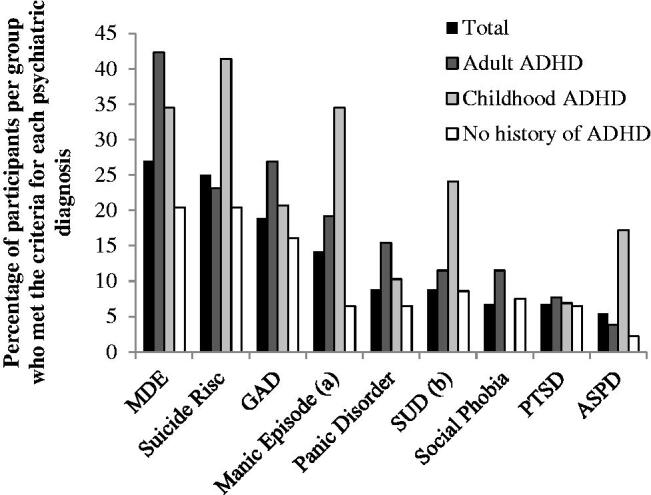
Psychiatric comorbidities of the total sample and by ADHD screening status. *Note*. This figure describes the current psychiatric comorbidity (mood, anxiety, and substance use disorders, and antisocial personality disorder) of the total sample (*N* = 136) by attention deficit/hyperactivity disorder (ADHD) screening status (no history of ADHD: *n* = 87; childhood ADHD only: *n* = 26; adult ADHD: *n* = 23). MINI = Mini International Neuropsychiatric Interview; MDE = major depressive episode; GAD = generalized anxiety disorder; SUD = substance use disorder; PTSD = posttraumatic stress disorder; ASPD = antisocial personality disorder. (a) Current and/or in the past, (b) other than alcohol use disorder.

Overall, 88 participants (64.7% of the sample) screened positive for at least one current psychiatric comorbidity other than ADHD. Figure 1 depicts current psychiatric comorbidities of the total sample and by ADHD screening status. Descriptively, participants with a history of ADHD symptoms in childhood and those who reported symptoms indicative of persistent ADHD had more psychiatric symptoms than those without a history of ADHD.

Table 2 indicates bivariate correlations between adult ADHD symptoms, childhood ADHD symptoms, and number of current psychiatric diagnoses. There was a significant association between ADHD screening status and number of psychiatric comorbidities, *F*(2,133) = 8.23, *p* < .001, partial *η*^2^ = .110. Participants who had no history of ADHD had significantly fewer comorbidities than those with a history of childhood ADHD symptoms (mean difference = 1.02, *p* < .001).

### Multiple mediation

**Figure 2.**
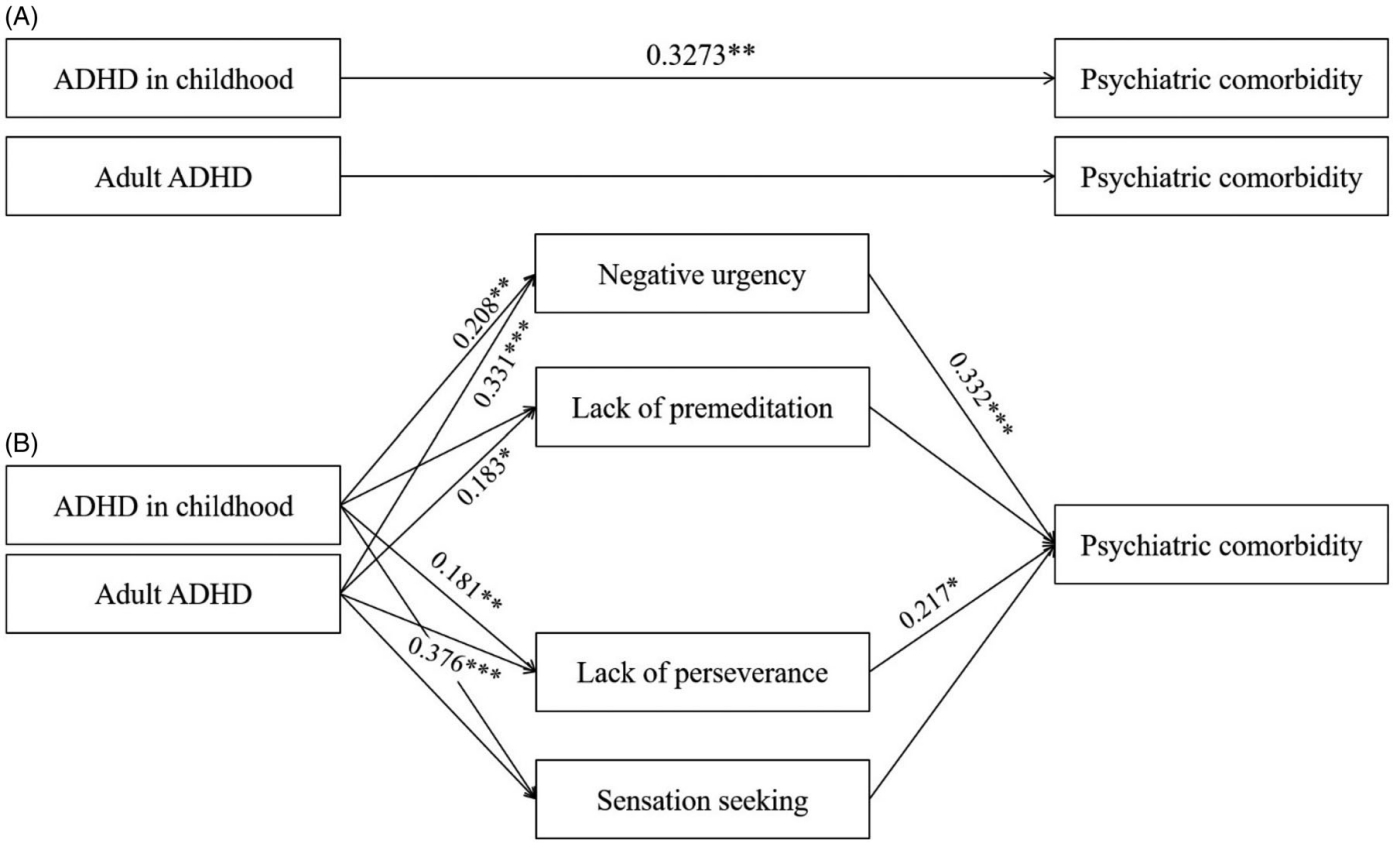
Multiple mediation model. *Note.* Completely standardized effect size indexes (*β*) are provided along significant paths. A denotes total effect of attention deficit/hyperactivity disorder (ADHD) screening status on psychiatric comorbidity (number of current psychiatric disorders, including mood and anxiety disorders, substance use disorders other than alcohol use disorder, and antisocial personality disorder; without mediators). B denotes multiple mediation model of ADHD screening status on psychiatric comorbidity via pathways to impulsive behavior. *p < .05, **p < .01, ***p < .001.

We tested whether impulsivity mediated the relationship of ADHD screening status on psychiatric comorbidity. There was a significant total effect of ADHD symptoms in childhood on psychiatric comorbidity (*β* = 0.327, 95% CI [0.498, 1.546], *p* < .001; Figure 2, Panel A) and a significant direct effect (*β* = 0.224, 95% CI [0.048, 1.371], *p* < .038; result not shown). This indicates that impulsivity partially mediated the effect of a history of ADHD symptoms in childhood on psychiatric comorbidity (Figure 2).

Table 4 reports all indirect effects of the individual mediators on the relationship of ADHD screening status to psychiatric comorbidity. There were indirect effects of both the negative urgency and lack of perseverance subscales (Table 4). Even though there was no significant total effect (*β* = 0.161, 95% CI [0.016, 1.071], *p* = .057) or direct effect of adult ADHD symptoms on psychiatric comorbidity (*β* = −0.013, 95% CI [−0.540, 0.526], *p* = .869; results not shown), there were indirect effects of both negative urgency and lack of perseverance subscales; i.e., 95% bias-corrected confidence intervals (CIs) for indirect effects of mediating variables did not contain zero (Figure 2; Table 4).

**Table 4.** Indirect effects of UPPS subscales.

Independent variable	Mediating variable	Dependent variable	*β*	Standard error	95% CI
ADHD symptoms in childhood ⇒	Negative urgency	⇒ Psychiatric comorbidity	1.9986	0.10820	0.04326, 0.46100
	Lack of premeditation		−0.3709	0.04952	−0.13662, 0.06091
	Lack of perseverance		1.6430	0.07455	0.00243, 0.29663
	Sensation seeking		0.0540	0.03535	−0.07289, 0.08281
Adult ADHD symptoms ⇒	Negative urgency	⇒ Psychiatric comorbidity	2.6054	0.13842	0.14678, 0.67909
	Lack of premeditation		−0.7070	0.08461	−0.25906, 0.06554
	Lack of perseverance		1.7923	0.14924	0.01292, 0.61252
	Sensation seeking		0.0624	0.04035	−0.08416, 0.08626

*Note.* ADHD = attention-deficit/hyperactivity disorder; CI = confidence interval.

## Discussion

The current study examined the role of impulsivity-related personality traits in explaining the relationship between an ADHD screening status and psychiatric comorbidity in Austrian patients with severe AUD. Our results demonstrated that almost half of the patients with childhood ADHD symptoms continued to have symptoms into adulthood (23 with adult persistent symptoms/49 with childhood symptoms). In concordance with previously raised concerns (Levin, 2007), only five participants had ever received any treatment for their ADHD, and only two of those had received pharmacological ADHD treatment.

Previous studies from Austria, using the same ADHD assessment instruments as the current study, reported a higher frequency of childhood ADHD symptoms but similar adult ADHD symptoms among prisoners with opioid use disorder (childhood ADHD: 50%, adult ADHD: 17%; Silbernagl et al., 2019) and treatment-seeking problem gamblers (childhood ADHD: 43%, adult ADHD: 11%; Brandt & Fischer, 2019). A study in German AUD inpatients reported a similar rate of retrospective childhood ADHD symptoms (20%) compared to our sample but a higher adult ADHD symptom rate (33%; Ohlmeier et al., 2008).

More than two third of the sample had at least one additional psychiatric disorder (other than AUD and ADHD). Consistent with the literature, the most common psychiatric comorbidities were major depression, manic episodes, anxiety disorders, and suicide risk (Grant et al., 2004; Melchior et al., 2014; Sørensen et al., 2018). In addition, patients with childhood ADHD symptoms had significantly more current comorbid psychiatric disorders than those who did not have a history of ADHD (Daurio et al., 2018; Ercan, 2003; Ibrahim et al., 2015; Moura et al., 2013; Young et al., 2020). This difference has been shown to be even more pronounced in active substance using populations with ADHD (Levin et al., 1998; van Emmerik-van Oortmerssen et al., 2014).

Impulsivity, and particularly negative urgency and (lack of) perseverance, mediated the relationship between childhood ADHD symptoms and psychiatric comorbidity. These results are in line with findings in individuals with gambling disorder (Grall-Bronnec et al., 2012), highlighting the role of specific impulsive personality traits in increasing the overall psychiatric burden among AUD patients with a history of ADHD. A strong association between urgency and problematic alcohol use has been shown in previous studies, most consistently among adolescents and young adults (Coskunpinar et al., 2013; Stautz & Cooper, 2013), and in studies examining the use of other substances such as cocaine and cannabis (Albein-Urios et al., 2012; Fernández-Serrano et al., 2012; Torres et al., 2013; VanderVeen et al., 2016). This link may be explained by succumbing to immediate impulses (e.g., to use a substance) in an attempt to downregulate intense negative emotions (despite negative consequences) among individuals who experience problems in emotion regulation (e.g., those who have ADHD) (Tice et al., 2001).

Less attention has been paid to perseverance, albeit a strong association between drinking quantity and lack of perseverance has been found (Coskunpinar et al., 2013). Alcohol use and lack of perseverance have been found to moderate the relationship between depressive symptoms and suicide proneness among college students (Dvorak et al., 2013). Thus, alcohol use in combination with this specific impulsivity trait may increase the association between negative emotional functioning and maladaptive outcomes. Given that depression and suicide risk were prevalent in the present sample, our results may suggest a similar pattern in that perseverance is a pathway of the relation between ADHD symptoms and psychiatric comorbidity. In addition, recent research in gambling disorder patients indicated that lack of perseverance predicted treatment dropout (Mallorquí-Bagué et al., 2019; Mestre-Bach et al., 2019). Lack of perseverance, reflecting a tendency to not persist in activities that can be complex such as a pharmacological/behavioral treatment schedule, may negatively impact treatment adherence and retention. Therefore, it may be related to a worse prognosis of the primary and comorbid diagnoses.

Interestingly, there was no significant total or direct effect of adult ADHD symptoms on psychiatric comorbidity. However, negative urgency and (lack of) perseverance indirectly affected the relationship between adult ADHD symptoms and psychiatric comorbidity. Previous research found similar results with regard to adult ADHD symptoms of impulsivity/emotional liability and AUD severity, highlighting the indirect effects of ADHD on AUD severity through unique facets of impulsivity (Daurio et al., 2018). The authors suggested that general symptoms related to emotional dysregulation and impulsivity may not by themselves exacerbate the severity of AUD, but that it is the combination of adult ADHD and the tendency to act impulsively in response to intense emotions that increase the likelihood of developing (more severe) AUD. Our findings expand these results by providing support for an emotional impulsivity risk pathway, whereby negative urgency and (lack of) perseverance partially accounted for the association between ADHD symptoms and psychiatric comorbidity.

Participants reported only mild to moderate levels of problem severity in the different HRQL dimensions and perceived their current overall health as relatively good. In addition, there was no association between ADHD symptoms and severity of HRQL problems or perceived overall health. It has been shown that patients with ADHD have low self-perception and self-awareness (Manor et al., 2012) and tend to underreport their symptoms (Sibley et al., 2012). This is even more pronounced in patients with a long substance use history (Piñeiro-Dieguez et al., 2016). Thus, AUD patients with comorbid ADHD symptoms may have under-reported their health problems. In addition, impaired HRQL may normalize during periods of abstinence (Sizoo et al., 2010), and given their inpatient status, all participants in this study were currently abstinent.

Our results provide further support for paying close attention to the expression of specific impulsive personality traits, particularly low perseverance and high emotion-driven impulsivity, in individuals with SUD and ADHD. This differentiated view on impulsivity in research may be particularly fruitful to decipher the underlying mechanisms of the association between AUD/SUD-ADHD comorbidity with increased addiction severity, more psychiatric diagnoses, increased suicide risk, and poorer treatment outcome/response (Heinz et al., 2015; Hershberger et al., 2017). Such findings may support clinicians in improving the prediction of psychopathology and corresponding risk behaviors (Berg et al., 2015; Coskunpinar et al., 2013). Studies with larger samples are tasked with examining if the symptomatic overlap between ADHD and specific comorbid conditions (e.g., ASPD or MDD) is related to different facets of impulsivity. In addition, future intervention research should explore differential change in impulsivity in AUD/SUD patients, and particularly those with comorbid ADHD, as a function of behavioral and/or pharmacological treatment to inform new or refined models of care (Um et al., 2018). For non-persistent, highly distractible patients (i.e., those characterized by a lack of perseverance), stimulant medication combined with cognitive behavioral therapy may help them maintain focus. In addition, negative urgency characterizes behaviors that may be successfully addressed with behavioral interventions that target emotion regulation (Waxmonsky et al., 2008; Zapolski et al., 2010).

Our results should be interpreted considering some limitations. First, mediation requires temporal precedence from the independent variable to the mediator to the dependent variable, and the mediator is presumed to cause the outcome (MacKinnon et al., 2007). Given the cross-sectional nature of the study, our mediator models were specified based on theory. ADHD is a developmental disorder and childhood symptoms were explicitly assessed with the WURS-k, whereas the UPPS captures the adult expression of impulsivity rather than the developmental trajectory. Therefore, even though impulsivity is a lower-level personality process that may precede an ADHD diagnosis (Kotov et al., 2017), we chose to model ADHD as the independent variable. Nonetheless, the cross-sectional assessment prevents the conclusion that specific aspects of impulsivity are a causal pathway between ADHD symptoms and psychiatric comorbidities. Second, some authors have questioned the specificity of the ASRS-v1.1 in substance using populations (Chiasson et al., 2012), while others suggested potential under-reporting of adult ADHD symptoms among patients in long-term residential treatment with a low drop-out rate (Luderer et al., 2019). Likewise, concerns have been raised regarding the WURS-k’s specificity (McCann et al., 2000; Ward et al., 1993). At the same time, DSM-5 diagnosis of adult ADHD only requires the presence of some symptoms prior to the age of 12, and not necessarily a full childhood ADHD diagnosis. Thus, it is possible that the rate of individuals having “true” adult ADHD in our sample is lower *or* higher than the rate we estimated using the WURS-k in combination with the ASRS-v1.1. A new ASRS version for DSM-5, though currently only available in a few languages (excluding German), showed good operating characteristics (Ustun et al., 2017) and may help circumvent sensitivity and specificity issues with screening scales in future research and clinical practice. Lastly, due to the lack of a validated German UPPS-P version, we were not able to assess self-reported behaviors arising from positive mood states (positive urgency). However, a recent meta-analysis questioned the conceptual and practical separability of the urgency scales by demonstrating patterns of correlations between positive urgency different psychopathologies similar to negative urgency (Berg et al., 2015).

In conclusion, this study suggests that the subcomponents of impulsivity such as impetuous reaction when experiencing negative emotions (negative urgency) and the tendency to not persist in activities (lack of perseverance) contribute to the relationship between ADHD symptoms (particularly those present in childhood) and psychiatric comorbidity among patients with severe AUD. An increasing focus on impulsive personality traits, in addition to diagnosing ADHD, may provide a more precise characterization of SUD patients and help guide individually tailored treatment decisions.
